# App-based learning for kindergarten children at home (Learning4Kids): Study protocol for cohort 2 and the school assessments

**DOI:** 10.1186/s12887-022-03737-w

**Published:** 2022-12-09

**Authors:** Frank Niklas, Efsun Birtwistle, Astrid Wirth, Tina Schiele, Anna Mues

**Affiliations:** Department of Psychology, Ludwig-Maximilians-University Munich (LMU), Leopoldstraße 13, 80802 Munich, Germany

**Keywords:** Home learning environment, Family intervention, Tablet-based learning, Kindergarten and school children, Home literacy environment, Home numeracy environment, Development of numeracy and literacy competencies, Digital learning, Mobile sensing, School assessments

## Abstract

**Background:**

Children’s early literacy and mathematical competencies are very important predictors for their later success in school and their educational attainment in general. However, not all children are able to develop to their full potential and some are at risk of failing to reach sufficient competence levels. The project “App-based learning for kindergarten children at home” (Learning4Kids) is designed as a longitudinal intervention study that tests the potential impact of a computer tablet-based intervention for kindergarten children and their families before school entry. Here, the focus lies on both, potential short-term and long-term influences on children’s competencies development in kindergarten and school.

**Methods/design:**

Learning4Kids uses a multi-method intervention approach and draws on expertise from different fields such as psychology, education, informatics, and didactics. It combines child test assessments with parental, educator, and teacher surveys and checklists, interviews as well as observations in the families to measure child competencies and their behaviour, and to assess family characteristics. The participating children and their families will be visited and assessed altogether seven times, starting in the second-last year of kindergarten until children are at the end of Grade 2. In cohort 1, 190 families participated in this project, whereas in cohort 2 another 310 families joined the Learning4Kids project. For the school assessments, standardized and curriculum-based tests will be used to assess children’s mathematical and literacy competencies. In addition, cognitive and non-cognitive child abilities will be assessed.

**Discussion:**

Learning4Kids offers substantive advances for the scientific fields of psychology and education, and also provides implications for policy and practice in the long term. Improving young children’s learning trajectories and analysing these trajectories from kindergarten to primary school is both a social and economic imperative as it contributes to greater individual success and thus to societal prosperity.

## Background

The development of children’s literacy and mathematical competencies is a continuous process that starts well before children enter school and precursor abilities in kindergarten children are meaningful predictors of academic performance in school (e.g., [1–3]). As early interventions are more cost effective and usually more successful [4], interventions aiming at supporting children’s competencies development should be implemented before school entry. Here, the family provides a context in which young children spend most of their time and in which interventions have been successfully applied (e.g., [5–7]).

As both, early and later mathematical and literacy competencies are important for children’s educational attainment, assessment tools need to be identified that measure these competencies reliably. Further, variables that are associated with these competencies such as children’s intelligence, families’ socioeconomic status (SES) and migration background and the quality of the home learning environment (HLE), should also be considered (e.g., [1, 8]). However, migration background and SES seem to impact on students’ academic achievements rather indirectly and various mechanisms are discussed that may explain this indirect association such as differences in living conditions, stress factors, parenting, and the HLE [9]. In particular, the HLE plays an important role for children’s early numeracy and linguistic competencies (e.g., [10, 11]) and child behaviour (e.g., [12, 13]).

### Digital family interventions

In many countries worldwide, children grow up in media-rich homes and are in daily contact with digital tools [14]. Consequently, utilizing these tools for the support of children’s competencies’ development seems to be promising. In fact, research indicates that interventions with digital tools can support children’s learning (e.g., [15, 16]).

Here, interventions were successful in supporting children’s behavioural outcomes [15], their mathematical competencies [16, 17], and their literacy competencies [18]. Moreover, these interventions were successful in laboratory settings [15, 16] as well as in naturalistic contexts such as in the family [19]. This effectiveness is partly due to the entertaining power of digital games that in these cases serve an educational purpose [20].

However, among the vast number of available learning applications (‘apps’) for digital devices, many apps do not provide appropriate and meaningful learning for young children [21, 22]. Consequently, it is important to identify and use specific criteria for the evaluation of existing learning apps as well as for the development of new apps that should lead to deep learning. Here, Hirsh-Pasek and colleagues [22] developed a framework with the four pillars of active, engaged, meaningful, and social interactive learning that a good learning app should provide, and they applied this framework to evaluate various often-downloaded apps [23]. While some of these apps were of high quality, many apps did not reach high quality levels, which signals the need for the development of more high-quality apps that support children’s deep learning.

### Rationale for the study and study aims

We know that a substantial number of children, and here in particular children who grow up in more difficult conditions, may not receive the kind of support at home that would be needed to develop to their full potential [24]. Therefore, it is important to develop effective, easy-to-apply intervention approaches that may support these children in their learning trajectories. As early literacy and mathematical abilities are the best predictors for later achievement in school [1–3, 25], it is expedient for interventions to focus on both, children’s early and later literacy and mathematical competencies. Technical devices such as computer tablets are now available in the majority of European households [14], and thus learning apps offer a great opportunity to provide such interventions.

However, often intervention studies focus on short-term development only and do not conduct follow-up assessments to test for potential long-term effects [20]. Consequently, one aim of this project is to implement an innovative computer tablet intervention and to test not only for potential short-term, but also for potential long-term impact on children’s literacy and mathematical competencies. This randomized-control intervention project will thus analyse the development of children’s competencies in the years prior to and after school entry (see also [26] for a more comprehensive overview of the specific project objectives).

## Methods/design

**Fig. 1:**
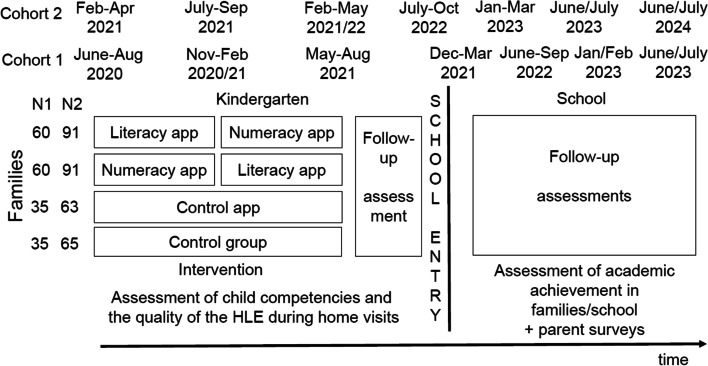
Schedule and timeline of the complete Learning4Kids project (first and second cohort)

Learning4Kids originally aimed at following 500 children and their families across 4 years from kindergarten until the end of grade 2. However, due to the Covid-19 pandemic and its impact on recruitment, we had to apply a two-cohort design instead. Here, a second cohort started a year later than cohort 1 and followed the same procedure as can be seen in Fig. 1.

All information about cohort 1 (sample, methods, test instruments), the intervention approach and the general study design can be found in study protocol 1 [26]. In the following, we will describe cohort 2, deviations in the assessments from cohort 1, and we focus on the assessment tools used in the later assessments and, in particular, during the school assessments.

### Sample

The second cohort of our sample consists of *N* = 310 children with their families. Here, *n* = 91 families were randomly assigned to each of the two experimental groups with a focus on literacy and numeracy learning. In addition, *n* = 65 families were randomly assigned to the control group without tablets and *n* = 63 families were randomly assigned to the control group with tablets (see Fig. 1).

Almost all participating families were recruited with the support of the Government Department administration office of Munich, which provided contact details of 6000 parents in the greater Munich metropolitan area who had children in their penultimate kindergarten year. We focused on regions in Munich with a higher ratio of families with a migration background (i.e., the child or at least one of the parents were born abroad) and with higher rates of low income and unemployment according to municipal demographic statistics [27]. The parents received a comprehensive invitation letter and a post card with the key information about the study. Interested parents were asked to get in contact with a team member via telephone or email. Here, parents were able to ask questions and research assistants were able to plan their first family visits and child assessments which took place from February to April 2021.

At the beginning of the first meeting, parents were provided with plain language statements about the project and with consent forms. These documents were prepared in the most commonly used foreign languages of the families in our sample (e.g., Turkish, Polish, Russian, Albanian, Romanian, Arabic, Persian (farsi), Spanish, Italian, Vietnamese, and English). After families signed the consent form, the children were also asked for their consent before the start of the assessments. In addition, a few families were recruited by word of mouth via participating parents and kindergartens in Cohort 1. Here, the consent forms were handed out and signed before the first family visit.

In Germany, almost all children of this age attend kindergarten 5 days a week for several hours a day [28]. Consequently, children’s educators were also contacted via telephone calls and mail, and asked to sign consent forms and fill in the surveys.

The *N* = 224 kindergartens in the project were attended by *N* = 1 to *N* = 6 study children (and their parents), while most kindergartens in the project were attended by one study child only. Descriptive statistics for all study groups are presented in Table 1.

**Table 1:** Descriptive statistics for the four groups of participating families in cohort 2

	Tablet group 1 (Literacy)	Tablet group 2 (Numeracy)	Control group 1 (Control tablet)	Control group 2 (No tablet)	Total sample
Number of participants, *N*	91	91	63	65	310
Children’s age at t1 in months, *M (SD),* range	59.0 (3.8) 51–67	59.7 (4.2) 53–69	59.4 (3.6) 52–67	59.3 (4.2) 53–69	59.4 (3.9) 51–69
Children’s gender: male / female	47 / 44	40 / 51	28 / 35	35 / 30	150 / 160
Families with migration background^a^, *N* (%)	33 (36.3)	45 (49.5)	22 (35.5)	26 (40.0)	126 (40.8)
SES^b^, *M (SD)*	107.4 (34.4)	108.1 (37.0)	106.28 (29.7)	104.63 (35.4)	106.8 (34.4)

^a^migration background: At least one parent or the child being born outside of Germany;

^b^SES: highest family occupational prestige [29]

The four groups did not differ significantly concerning any of the descriptive statistics. Further, similar to cohort 1, the ratio of families with a migration background was higher than in the general population of this age group [30]. Compared to cohort 1, children in cohort 2 were 4 months younger at t1, which is not surprising as the assessments were conducted a year later but about 4 months earlier within the kindergarten year. In cohort 2, the average highest occupational prestige score in a household was higher compared with other studies with medium SES samples (e.g., [5]), which indicates that despite the aim to recruit families with mixed socioeconomic backgrounds, a higher ratio of families with high SES decided to participate in this study.

The same descriptive statistics for the total sample (cohort 1 and cohort 2 combined) is shown in Table 2. Here, also no significant differences across the four groups were found concerning child age, sex, migration background and SES.

**Table 2:** Descriptive statistics for the four groups of participating families in the total sample (cohort 1 and cohort 2 combined)

	Tablet group 1 (Literacy)	Tablet group 2 (Numeracy)	Control group 1 (Control tablet)	Control group 2 (No tablet)	Total sample
Number of participants, *N*	151	151	98	100	500
Children’s age at t1 in months, *M (SD),* range	60.8 (4.4) 51–73	61.0 (4.5) 53–75	60.9 (4.7) 51–73	61.3 (5.0) 53–73	61.0 (4.6) 51–75
Children’s gender: male / female	76 / 75	71 / 80	45 / 53	51 / 49	243 / 257
Families with migration background^a^, *N* (%)	60 (40.0)	69 (45.7)	39 (40.2)	42 (42.0)	210 (42.2)
SES^b^; *M (SD)*	102.2 (37.5)	101.5 (37.8)	101.1 (34.2)	98.4 (35.5)	101.0 (36.5)

^a^migration background: At least one parent or the child being born outside of Germany;

^b^SES: highest family occupational prestige [29]

### Measures

Assessments in Learning4Kids included educator and parental surveys and checklists, observations and interviews in the family, and child test assessments (see [26]). An overview of the child test assessments for the complete study duration can be seen in Table 3.

**Table 3:** Overview of the child assessments in Learning4Kids

		t1	t2	t3	t4	t5	t6	t7
Mathematical competencies	MARKO-S (early mathematics)	X	X	X	X	X C2; C1_K	X K	-
	WVT; number sequence forwards	X	X	X #	X #	X #; C2; C1_K	X K #	-
	WVT; number sequence backwards	X	X	X	X #	X #; C2; C1_K	X K #	-
	WVT; predecessors and successors	X	X	X	X #	X #; C2; C1_K	X K #	-
	WVT; Numerical knowledge	X	X	X #	X #	X #; C2; C1_K	X K #	-
	MBK-0 (calculating task)	X	X	X #	X #	X #; C2; C1_K	X K #	-
	HRT (addition, substraction)	-	-	-	X # C1	X #	X	X
	DEMAT (formal mathematics)	-	-	-	-	X C1_S	X S	X
Linguistic competencies	EuLe 4-5 (early literacy)	X	X	X	-	-	-	-
	PPVT-4 (passive vocabulary)	X	X	X	X	X C2; C1_K	X K	-
	WVT; rhyming task	X	X	X #	X #	X #; C2; C1_K	X K #	-
	WVT; letter sound identification	X	X	X #	X #	X #; C2; C1_K	X K #	-
	WVT; productive letter knowledge	X	X	X #	X #	X #; C2; C1_K	X K #	-
	WVT; receptive letter knowledge	X	X	X #	X #	X #; C2; C1_K	X K #	-
	SETK 3-5 (plural forms)	X	X	X	X	X C2; C1_K	X K	-
	BAKO 1-4 (phonological awareness)	-	-	-	-	X C1_S	X S	X
Active Vocabulary	AWST (verbs and nouns)	X	X	X	-	-	-	-
	WWT (verbs, nouns, categories, opposites)	-	-	-	X	X	X	X
Reading & Spelling	WLLP (Reading)	-	-	-	X C1_S	X S	X C1; C2_S	X
	DERET (Spelling)	-	-	-	-	X C1_S	X S	X
	ELFE (Reading comprehension)	-	-	-	-	-	-	X
Intelligence	CMMS	X	X	-	-	-	-	-
	CFT 1-R (subtests 3 & 5)	-	-	X	X	X	X	-
	CFT 20-R (subtests 1.3 & 2.2)	-	-	-	-	-	-	X
Concentration	KKA	X	X C1	-	-	-	-	-
	FTF-K	-	X C2	X #	X #	-	-	-
	WISC-4 (concentration subtest)	-	-	-	-	X	X	X
Working memory	WVT (short term memory)	X	X	X	X			
	WISC-4 (working memory)	-	-	-	-	X	X	X
Rapid naming	-	X	X	X	X	X K	X K	-
Self-concept	BSLS	X	X	X	X	X K	X C2_K	-
	SRK	-	-	-	-	X S	X C1; C2_S	X
Prosocial behaviour	-	X	X	X	-	-	-	-
Need for Cognition	NFC-Kids	-	-	-	X	X	X	X

Note: X = applied; - = not applied; K = in kindergarten; S = in school; C1 = cohort 1, C2 = cohort 2; # = adapted test version

#### Test instruments

Several test instruments are used to assess various child competencies (information about all tests used at t1 can be found in Niklas et al. 2020 [26]). During the project period, some of these assessment tools needed to be adapted or substituted as children grew up, became more competent and thus ceiling effects were found. In the following, deviations from the original tests conducted at t1 will be described, before information about the test instruments that will be used in the later assessments and in school are presented.

1. Deviations from the original child test assessments at t1

Due to ceiling effects in some of the child test instruments, some assessments were adapted and new items were added (see Table 3, adapted tests are indicated with #). These additional items were presented to children only if they were able to solve all or all but one of the original items correctly to prevent children with lower abilities from getting frustrated.

For number sequence forwards and numerical knowledge (WVT), four additional items and for number sequence backwards and predecessors and successors (WVT) two additional items were added. Here, larger numbers were used for this task and the most difficult items were beyond the number 100 in each of these subtests. Similarly, in the calculation task (MBK-0), three additional items were included that comprised sums and subtractions up to 21.

The early literacy task (EuLE 4–5) was very easy for children at t3 and was omitted from further assessments. The rhyming and the letter sound identification tasks (WVT) were adapted and additional items comprising more difficult words and non-words were added. As ceiling effects were also found for productive and receptive letter knowledge (WVT), here also additional letters and finally easy words to be identified were added. All these adapted tests assessing children’s mathematical and linguistic competencies as well as the tests that were not adapted such as the MARKO-S and the SETK 3–5 (see [26]) will be kept until the end of t4 assessments (end of t5 assessments for cohort 2), except for the children with a delayed school entry who will be tested with these tests until the end of t6 which is at the end of their prolonged kindergarten attendance.

Further, vocabulary was assessed at t1 to t3 with the AWST-R task. However, due to ceiling effects, this assessment was substituted with the “*Wortschatz- und Wortfindungstest für 6- bis 10-Jährige”* WWT [31]. Here, instead of verbs and nouns only, also categories and word opposites were tested. For categories, children were presented with 4 pictures on a page and were asked what these pictures have in common (e.g., when four different animals were presented, the common category to be named would be “animal”). Word opposites were tested by first explaining to children what this term means and then asking them specific questions such as “what is the opposite of small”. Altogether 20 items (5 nouns, 5 verbs, 5 categories, and 5 opposites) were presented to the study children.

2) Formal mathematics

To assess children’s knowledge of formal mathematics, two assessment tools will be used. First, a short version of the addition and subtraction subtests of the *“Heidelberger Rechentest (HRT 1-4)”* [32] will be implemented at t4 for cohort 1 and at t5 for both cohorts. The HRT subtests give a differentiated and reliable (retest reliability for retesting after 2 weeks r_tt_ = .82 and .86, [32]) overview of children’s mathematical basis calculation abilities. For later assessments, the complete subtests will be used for the children attending school.

Second, the “Deutscher Mathematiktest” DEMAT1+ [33] will be used at the end of Grade 1 and the DEMAT2+ [34] will be used at the end of Grade 2 to assess children’s mathematical competencies. These tests are standardized and curriculum-based and thus designed to measure children’s mathematical school abilities very well. All nine subtests of the DEMAT1+ address different content areas including number line tasks, linkage of numbers to quantities, addition and subtraction, number decomposition and completion, number chain tasks, and written mathematical problems. This test has a good reliability with Cronbach’s α = .90 [33]. The ten subtests of the DEMAT2+ include addition and subtraction, comparison of lengths, written mathematical problems, geometry, division and calculating with money. The reliability of DEMAT2+ is very good with Cronbach’s α = .93 [34].

3) Reading and spelling

Advanced phonological awareness as a predictor of reading and spelling will be assessed with two subtests of the "Basiskompetenzen für Lese-Rechtschreibleistungen" (BAKO 1-4) [35]. In one subtest, the children were asked to substitute each vowel "a" with the vowel "i" in a given word, whereas in the second subtest, they were asked to swap the first two phonemes in a given word. The authors report a high reliability for this test (Cronbach’s α = .90-92). Early reading will be assessed with the “Würzburger Leise Leseprobe – Revidierte Version” (WLLP-R) [36]. Here, children are expected to read a word next to four related pictures in a row. Then, they need to decide which picture aligns best with the word they have read and are expected to mark this word. Here, the instruction for children is to read and mark the words as fast as they are able to within a given time. Consequently, the WLLP-R measures children’s speed of decoding words when reading. The authors report a high reliability of this test for first to fourth graders (*r* = .87–.93).

With the “Deutscher Rechtschreibtest für 1. und 2. Klassen” (DERET 1–2+, [37]) children’s spelling abilities will be assessed via short dictations. The text for the first graders consists of 29 words and the text for second graders comprises 52 words. The internal consistency of the DERET 1–2+ is high with Cronbach’s α = .89–.92, [37].

Finally, at the end of Grade 2, the “Ein Leseverständnistest für Erst- bis Sechstklässler” (ELFE, [38]) will be used to assess children’s reading comprehension. Here, children’s reading comprehension will be assessed with subtests on word, sentence, and text comprehension while taking the reading speed into account. The internal consistency of the subtests lies between Cronbach’s α = .92–.97 [38].

4) Other test instruments
  a Intelligence
    At t1 and t2, children’s general cognitive abilities were tested with the Columbia Mental Maturity Scale [39]. From t3 to t6, children’s intelligence will be assessed with two subtests of the “Culture Fair Test” (CFT 1-R) [40], a reliable measure of nonverbal intelligence (Cronbach’s α = .94–.95). In the classification task, children have to identify and select the one object out of five, which does not fit with the other ones. In the matrix task, children have to identify and select which object out of five fits in the empty box of objects shown. Here, not only the correct object needs to be found, but also the pattern and the order of the objects need to be considered. In the final assessment at t7, the CFT 1-R will again be substituted with the two subtests “classification” and “matrix” of the CFT 20-R for older children [41].
  b Concentration
    Due to ceiling effects, the concentration task used in cohort 1 in t1 and t2 [26] was substituted with another short concentration task, the “Frankfurter Test für Fünfjährige - Konzentration” (FTF-K) [42]. In cohort 2, this test was already used in t2. Here, children had to cross the pears among a page full of apples. As this task was also fairly easy for the study children, the total time of this task was reduced from 90 to 60 seconds. This test has a sufficient retest-reliability for short periods of time [43]. During the last three assessments, another test will be used to assess children’s concentration. In this subtest from the “Wechsler Intelligence Scale for Children – Fourth Edition” (WISC-IV) [44], children have to cross all the animals they can find on a large page with animals and objects in a random order within 60 seconds. The reliability of this concentration task for the age group is .76 [44].
  c Working memory
    After t4, the short-term memory test (see [26]) will be substituted with a working memory task from the WISC-IV [44] in which children hear a row of numbers which have to be repeated backwards. This task starts with two numbers up to a maximum of eight numbers in a row. The reliability of this working memory task for the age group is .78 [44].
  d Self-concept
    Once children enter school, a new assessment tool will be used to measure children’s mathematical, reading, and spelling self-concept [45]. Consequently, this scale assesses children’s academic self-concept and it had successfully been used in a large-scale longitudinal study with kindergarten and primary school children in Germany [46]. Children have to rate on a seven-point Likert-type scale with small stick figures representing their classmates, what they think about themselves in comparison to other children (e.g., “In reading, I am the worst/best”, “mathematics is no fun/ a lot of fun”). Here, children have to conduct social comparison processes and rate their own evaluative and emotional beliefs about their academic achievement. In addition, new items using the same scale were developed to also assess children’s prosocial and antisocial self-concepts. Similar to the assessment of the academic self-concept, children were asked to rate their potentially prosocial and antisocial behaviours and compare themselves with their peers.
  e Need for cognition
    From t4 onwards, a new assessment tool will be used to assess children’s need for cognition [47, 48]. Here, short statements such as “I like to think about solutions for problems” are read to children. Afterwards, they are asked to rate on a visual scale, how much each of these statements applies to them (“yes, that is always true”, “sometimes that’s true, but sometimes it’s not” or “No, that’s not so true.”). This tests measures children’s motivation and interest in cognitively challenging tasks. The total score “think” is reliable (Cronbach’s α = .86 [47]) and comprises the subscales “seek” and “conquer”.
  f Prosocial behaviour
    Here, the assessment at t1 was kept unchanged until t3 (see [26]) and afterwards this test was omitted from the assessment schedule.

#### Educator and teacher survey

Kindergarten educators were asked to fill in a survey on children’s characteristics. The educators rated which digital tools are available in the kindergarten and how often they are used. Further, the educators were asked to provide a general evaluation of children’s basic competencies such as concentration, linguistic, mathematical, social integration into the kindergarten group, and socio-emotional competencies. As reported in Niklas et al. [26], children’s emotional and social abilities were assessed with the “Entwicklungsbeobachtung und -dokumentation” (EBD) [49] and children’s behavioural strengths and difficulties with the “Strength and Difficulties Questionnaire” (SDQ) [50]. However, the items used will be adapted to the age group of the children during the study period. In the last year of kindergarten, educators are also asked about children’s school readiness. Further, in the school assessments, the items of the EBD will be substituted with respective items from the “Leipziger Kompetenz-Screening für die Schule” (LSK) [51] and teachers will be additionally asked about parent’s school involvement with four items (e.g., “The parents of the child participate in school activities, such as Christmas fairs or cake sales”).

#### Parental survey

As with the Plain Language Statements, parental surveys were provided in several languages addressing families with various migration and language backgrounds (see above). Parents were asked questions on their family background, children’s characteristics, children’s and parents’ media competencies and media usage at home, and the HLE [26, 52]. Here, the focus lies for all assessments on various aspects of the HLE, including questions on informal and formal parent-child interactions that support children’s literacy and mathematical learning as well as parental attitudes towards reading (e.g., “reading is an important activity in our home”) and mathematics (e.g., “mathematics is considered important in our home”).

In addition, two checklists were used to get further insight into the home literacy and numeracy environment. Here, a German book title recognition test for children’s books (TRT-VS [53]) and a German mathematical game titles recognition test (TRT-Mathe-K [54]) were applied. In these checklists, selected titles of children’s books and math games were mixed with fake book and game titles to objectively assess parents’ knowledge of these books and games.

Further, parents’ expectations for their child’s future school education (example item: “The education of my child in school is important to me”), children’s and parents’ media usage (example item: “Do you have learning apps/games for children on your tablet/smartphone? How often are these used?”), attitudes towards digital media (example item: “Digital media for preschool children are important”), and children’s and parental media literacy (example item: “How would you rate your ability to find appropriate media content for children?”) were assessed at t1 [26] and will be assessed throughout the project. Once children enter school, parents will also be surveyed about their children’s school experience (e.g., “my child likes to attend school”), about the homework situation (e.g., “I explain a task to my child, if he or she does not understand it”), and about parental school involvement (e.g., “I participate in parents’ evenings at my child’s school”) [55, 56].

In cohort 2, an additional parental survey assessing parents’ beliefs towards mathematics was included and both caregivers at home were asked to fill it in. Here, parents were asked about their own self-efficacy, gender stereotypes toward mathematics and literacy, and their beliefs and attitudes towards mathematical activities at home. Parents’ self-efficacy was measured with six items (e.g., “At school, I was good at math”; Mother’s Cronbach’s α = .75; Father’s Cronbach’s α = .77) [57–59]. Parental gender stereotypes were measured with six items including “Girls need less assistance than boys in mathematics” (Mother’s Cronbach’s α = .87; Father’s Cronbach’s α = .88) [57, 60]. Further, parents were asked with three items to evaluate the importance of their child doing mathematical activities at home (e.g., “It is important to me that my child does mathematical activities at home”; Mother’s Cronbach’s α = .50; Father’s Cronbach’s α = .62) [57]. These additional items were adapted for our study [61] and values from 0 to 4 were assigned, with 0 indicating “not true at all” and 4 indicating “completely true”.

#### Assessment of the HLE

There are different ways to assess the HLE and the debate about the most suitable assessment method is still ongoing (e.g., [62, 63]). To assess the HLE as comprehensive as possible in a large-scale longitudinal study, Learning4Kids uses several operationalizations such as parent surveys on the home literacy environment and the home numeracy environment (see above), observations of parent-child interactions during shared reading and playing a dice game, and children’s book title and mathematical game title checklists (for more detail, please refer to [8]). For the second cohort, two additional observation items were included that assess also the response behaviour of the child in addition to the quality of the parent-child interaction in general [64]. As both checklists (book titles and mathematical games) consist of two parallel forms, they are swapped at each subsequent assessment. In addition, for the later assessments in school, a book title checklist for older children (K-TRT, [65]) will be used instead of the checklist for younger children. Finally, once children are at the end of grade 1, parents will also be surveyed about children’s own reading behaviour (e.g., “At home, my child reads to me”).

#### Assessment of the app usage

To test for potential effects of an intervention, it is important to consider the intervention fidelity, that is, whether the intervention is delivered as intended by the protocols [66]. However, only a few large-scale education studies report on the fidelity of implementation of kindergarten or school curriculum interventions [67]. In Learning4Kids, we used a separate app to assess the usage times of each of the learning apps that were part of the intervention through mobile sensing technology [68], see also [26]. With this approach, the exact usage times for all learning apps on the study tablets were obtained and were used for analyses and for a reward system for the study children. Further, detailed intervention protocols and questionnaires for parents and children were used to check whether the participants had engaged with the intervention content. Consequently, our project followed the recommendations provided for good intervention fidelity [66].

### Analytic strategy

Given that this study includes comprehensive longitudinal assessments, numerous analyses are conceivable. Consequently, in addition to the testing for potential short- and long-term intervention effects on children’s literacy and mathematical competencies (see [26]), other research questions, for instance, concerning the home learning environment can be conducted (e.g., [8, 57]). Here, structural equation modelling can be used to analyse the complex longitudinal associations between the study variables and children’s development in the family context.

## Discussion

The main goal of the project “Learning4Kids” is to develop, implement and evaluate an easy-to-apply tablet intervention in the family context and to test for potential short- and long-term effects on children’s development of mathematical and literacy competencies. Given that many children grow up in environments that do not provide optimal educational support [24], such an intervention may impact on children’s learning trajectories as early literacy and mathematical abilities may be enhanced which, in turn, will predict later school achievement [25].

However, we are still in need of studies that not only analyse immediate post-intervention effects but also test for potential long-term effects. Such analyses are important to justify the implementation of costly long-term interventions. In case these interventions show immediate effects only, this information would also be very valuable as then interventions may need to be implemented for longer durations or even in an on-going fashion (e.g., with continuous learning content).

As this randomized-control intervention study will follow the study children from the second-last year of kindergarten until the end of Grade 2, it will add to our knowledge about potential long-term effects of kindergarten interventions. Consequently, we believe that this project has both, the potential to provide us with valuable scientific insight for future studies and to have a positive impact on children’s development.

## Data Availability

The raw data supporting the conclusions of this manuscript will be made available by the authors, without undue reservation, to any qualified researcher on reasonable request.

## Abbreviations

Apps: Computer tablet applications
AWST-R: Active vocabulary test for 3- to 5-year-old children
BAKO: Assessment of basic competencies for reading and writing (phonological awareness)
CFT 1-R: Basic intelligence test scale 1 – revision
CFT 20-R: Basic intelligence test scale 2 - revision
DEMAT: German mathematical test battery
DERET 1–-2+: German Spelling Test for Grade 1 and 2
EBD: Observation and documentation of children’s development 48–72 months
ELFE: A Reading Comprehension Test for Grade 1 to Grade 6
ERC: European Research Council
EuLe 4–5: Assessment of narrative and reading competencies of 4- to 5-year-old children
FTF-K: Concentration test
HLE: Home learning environment
HRT: Heidelberg Arithmetic Test for primary school children
K-TRT: Book title-recognition test for school children
Learning4Kids: App-based learning for kindergarten children at home
LMU: University of Munich
LSK: Leipzig competencies screening for schools
MARKO-S: MARKO-Screening – mathematics and concepts of calculation before school entry
MBK-0: Assessment of basic mathematical competencies in kindergarten age
PPVT: Peabody Picture Vocabulary Test
SDQ: Strengths and Difficulties Questionnaire
SES: Socio-economic status
SETK 3–5: Linguistic development test for three- to five-year-old children
TRT-Mathe-K: Mathematical game titles recognition test
TRT-VS: German book title recognition test for children’s books
WISC-IV: Wechsler Intelligence Scale for Children – Fourth Edition
WLLP: Würzburg silent reading test – revision
WVT: Würzburg preschool test: Assessment of literacy and mathematical (precursor) abilities and linguistic competencies in the last year of kindergarten
WWT: Vocabulary and word-finding-test for 6–10-year-olds
